# Supplementary figures and images for: Oral magnesium supplementation for leg cramps in pregnancy—An observational controlled trial
Source: PLoS One. 2020 Jan 10;15(1):e0227497. doi: 10.1371/journal.pone.0227497 (PMC6953803; doi:10.1371/journal.pone.0227497)

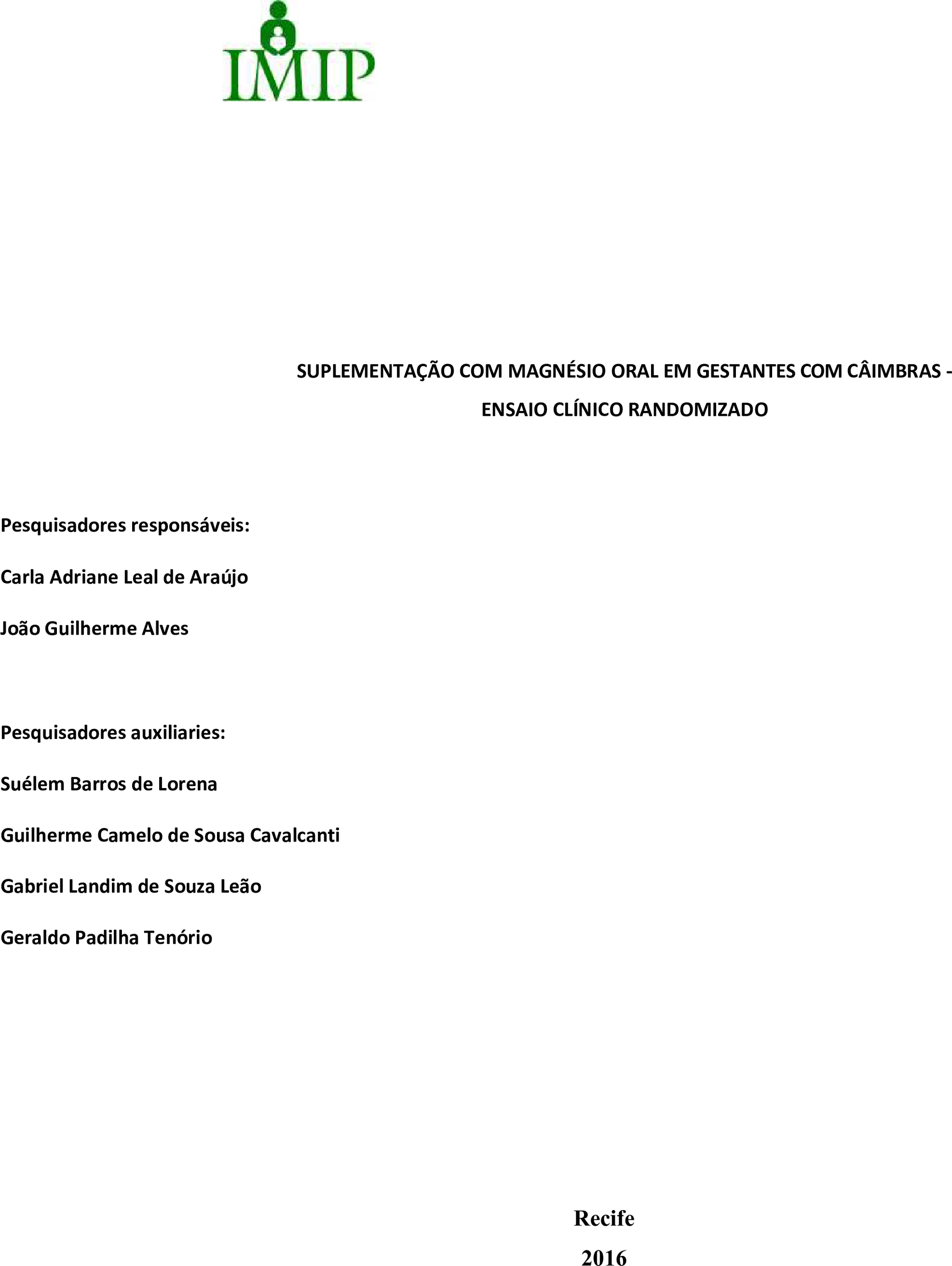

Supplement: S1 Fig — (TIF) [file pone.0227497.s001.tif]

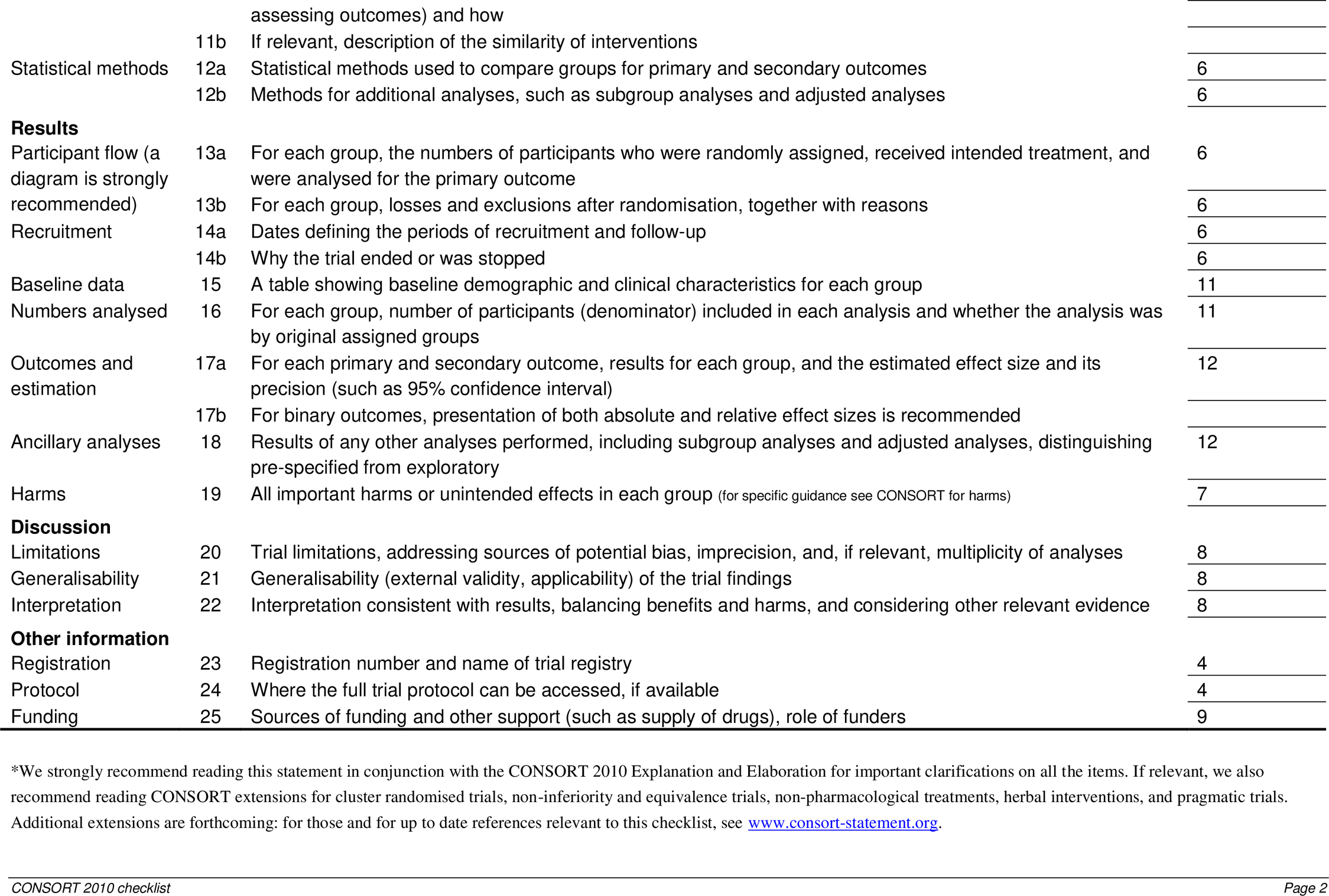

Supplement: S2 Fig — (TIF) [file pone.0227497.s002.tif]

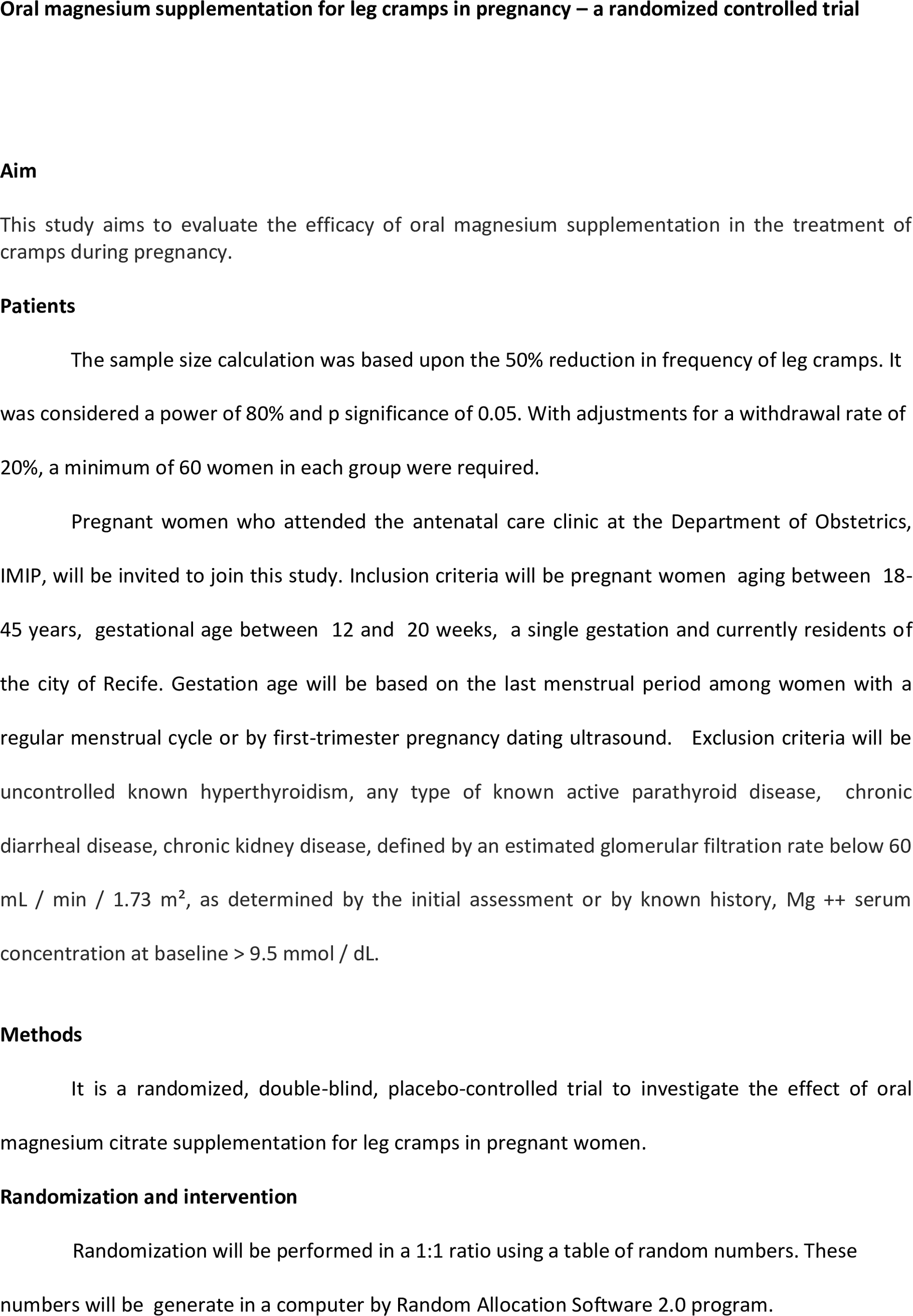

Supplement: S3 Fig — (TIF) [file pone.0227497.s003.tif]
