# Supplementary material for: Oral magnesium supplementation for leg cramps in pregnancy—An observational controlled trial
Source: PLoS One. 2020 Jan 10;15(1):e0227497. doi: 10.1371/journal.pone.0227497 (PMC6953803; doi:10.1371/journal.pone.0227497)
Supplement: Attachment — Submitted filename: Answers to the Reviewers (1).docx [file pone.0227497.s005.docx]

**Answers to the Reviewers:**

***Answer: Thank you very much for reviewing our manuscript. We also greatly appreciate the reviewers for their comments and suggestions. We have carried out all the recommendations suggested by the reviewers. The manuscript has been completely revised and we hope it will be able to be published.***

Reviewer #1: I will focus on methods and reporting

Major
1) the introduction is very short for a research paper. Much more information is needed in terms of the background to the study.

***Answer: The introduction was complemented. A new paragraph and five news references were added.***

2) I don't understand how the sizes of the groups differ so much following an 1 to 1 allocation process

***Answer: We apologize for this mistake. All the database was reviewed with a biostatistician and it was observed that eleven 18-year-old pregnant women had not been included in the analysis. These 11 pregnant women (2 in the intervention group and 9 in the control group) were now included in the analysis and the ratio 1:1 restored.***

3) the methods section is very poor. Power analyses are incomplete. What is the baseline level for cramps? power and numbers needed are very different between a very prevalent and a rare outcome. a 50% reduction is a a very large effect. What is the odds ratio? the analysis plan is pretty poor. Even if perfectly balanced why did they not analyse in the context of a logistic regression, where the effects could be quantified in ORs?

**Answer: Baseline level for cramps was included. The sample size calculation was based upon the 50% reduction in frequency of leg cramps in both groups obtained from Dahle et al (reference number 12) and Supakatisant C & Phupong V (reference number 13) trials. The odds ratio was calculated by logistic regression. The effects were now quantified in ORs.**

Minor
1) Abstract. 1 to 1 matching yes 50 vs 63 in the placebo group. that's not 1 to 1?

***Answer: It was corrected (66:66)***

2) information on the blinding is needed in title and/or abstract. single? double?

***Answer: It was provided; double blind.***

3) abstract says the 2 groups were comparable. some elaboration is needed

***Answer: It was added this information.***

4) no information on analyses methods in the abstract

***Answer: It was provided.***

5) no information on baseline levels in the abstract - only the reduction. we need some measure of the absolute, not only relative, reduction.

***Answer: This information was added in the abstract.***

6) Abstract: report effects and their CIs rather than p-values

***Answer: Effects and their CIs were provided.***

Reviewer #2: This is a randomized controlled trial to evaluate the effect of oral magnesium supplementation on leg cramps in pregnant women. There are many critical design flaws for this study. The data provided in the manuscript was not consistent in whole manuscript. Also, many writing issues and language questions presented in the manuscript.
I will list several important design flaws:
1. The ratio for randomization was 1:1, while the number of women in Mg supplementation group was 57 and 64 in the placebo group. The number of participants in two groups were imbalanced. And the author did not provide the detailed allocation concealment for randomization. This is very important in a RCT study. Without allocation concealment, randomization would be unsuccessful.

**Answer: *We apologize for this mistake. All the database was reviewed with a biostatistician and it was observed that eleven 18-year-old pregnant women had not been included in the analysis. These 11 pregnant women (2 in the intervention group and 9 in the control group) were now included in the analysis and the ratio 1:1 restored.* Allocation concealment was ensured, as the service did not release the randomization code until the participants were recruited into the trial after all baseline measurements were completed.**

2. The sample size calculation was based on the estimation of 50% reduction in frequency of leg cramp in Mg treatment group. The primary outcome was the presence of the leg cramps. The current sample size might not be sufficient for detecting the differences in the primary outcome, since they calculated it according to the frequency of leg cramp. Additionally, the 50% reduction in frequency of leg cramp in treatment group might be too positive. All these above might be the reasons why this study had a negative result.

**Answer: The sample size calculation was based upon the 50% reduction in frequency of leg cramps in both groups obtained from Dahle et al (reference number 12) and Supakatisant C & Phupong V (reference number 13) studies. This limitation was included in the discussion.**

3. The method for missing values were not mentioned in the study.

***Answer: Thank you for this observation. However as we had no missing values we did not include this in the methods section.***

Reviewer #3: 1. The hypothesis testing, power and type 1 error should be included in the study design section. Please also be clear whether the type 1 error is one-sided or two sided.

***Answer: The hypothesis testing, power and type error were included in the methods section.***

2. The screening failure rate is very high. Any reasons for this?

***Answer: The flowchart was corrected. The main reason for the screening failure was absence of leg cramps.***

Reviewer #4: In this RCT, oral magnesium did not reduce the occurrence nor frequency of leg cramp episodes in the second trimester of pregnancy. This is an interesting topic, but some details are missing from this manuscript.

1.) The rationale for the trial requires more explanation in the introduction to highlight any gaps in knowledge. For example, the Garrison et al. (2012) systematic reviewed showed no evidence that oral Mg is helpful for leg cramps during pregnancy, with low heterogeneity among trials. What will this RCT add?

***Answer: Garrison et al is cited in the introduction (reference number 11) and they final conclusions are that “It is unlikely that magnesium supplementation provides clinically meaningful cramp prophylaxis to older adults experiencing skeletal muscle cramps. In contrast, for those experiencing pregnancy-associated rest cramps the literature is conflicting and further research in this patient population is needed.” We studied pregnancy-associated cramps and our conclusion is the same of Garrison et al, i.e. it is unlikely that magnesium supplementation during pregnancy provides clinically meaningful cramp prophylaxis to leg-cramps.***

***This systematic review implications for research were: “To resolve the uncertainty surrounding the role of magnesium in pregnant women, parallel‐group blinded placebo‐controlled RCTs of magnesium in that population are needed”. We tried to develop a randomized double-blind controlled trial.***

2.) In the introduction, it would be more relevant to cite magnesium effects on muscle/ mechanistic effects rather than cite general information on the etiology of cramps.

***Answer: The introduction was complemented. A new paragraph and four news references were added.***

3.) How common are cramps during the second trimester (study population used in the RCT)?

***Answer: During the second trimester 70.0 % (91/130) of 130 participants that had leg cramps in the first trimester continued presenting leg cramps.***

4.) pg. 5: Exclusion of those with Mg at baseline >9.5mmol/dL is not physiologically plausible given the Mg reference range. this value is a typo?

***Answer: Thank you for this correction. It was a typo error and it was corrected (2.6 mg/dL)***

5.) Was adherence self reported?

***Answer: Compliance/adherence, adverse events, and clinical intercurrences were monitored by the research team at each routine prenatal visit until the completion of the treatment. Adherence was defined as the ingestion of at least 80% of the prescribed dose.***

6.) How were outcomes ascertained?

***Answer: All this informations were taken from the research diaries previously given to participants.***

7.) Additional statistical rationale for the sample size calculation is needed. Why was a 50% reduction in leg cramps expected?

***Answer:*** ***The sample size calculation was based upon the 50% reduction in frequency of leg cramps in both groups obtained from Dahle et al (reference number 12) and Supakatisant C & Phupong V (reference number 13) studies.***

8.) pg. 7: Lines 3-4: are units in mg/dL? Three serum levels are listed: should one be deleted?

***Answer: The units are in mg/dL. It was provided. The first serum level was deleted.***

9.) Provide information on whether any stopping rules were instituted (CONSORT missing item)

***Answer: The criteria for discontinuation of the study were: clinical signs or symptoms reported by the patient due to the intake of the capsules or the cancellation of prenatal care at hospital.***

10.) Who was blinded to the information? (eg. only participants, their doctors, the PI, those assessing outcomes, the statistical analyst, etc)?

***Answer: Participants, their doctors and all investigators. This information was now provided in the method section.***

11.) Table 1: income values are approximately $50,000 annually? Units need checking

***Answer: Thank you for this observation. It was corrected.***

12.) Flowchart (Fig 1): Indicate which arm is the placebo; which is the Mg arm (currently unlabeled)

***Answer: It was indicated***.

13.) Can the original Portuguese protocol be translated into English via an online translator or individual? The English summary of the protocol is an abbreviated version of the manuscript and provides no additional detail.

***Answer: The Portuguese protocol was translated into English.***

14.) The overall manuscript would benefit from copyediting before resubmission

***Answer: The manuscript was reviewed.***
