# Supplementary material for: Oral magnesium supplementation for leg cramps in pregnancy—An observational controlled trial
Source: PLoS One. 2020 Jan 10;15(1):e0227497. doi: 10.1371/journal.pone.0227497 (PMC6953803; doi:10.1371/journal.pone.0227497)
Supplement: Attachment — Submitted filename: Rebuttal Letter.docx [file pone.0227497.s006.docx]

**Rebuttal Letter**

**Dear Editor,**

***We appreciate your attention. Thank you very much for the opportunity to address the comments from the Reviewers. We carefully considered all comments offered by the reviewers. The authors hope that the Reviewers will be satisfied with the further amendments which we have made to the manuscript. Please see below the point-by-point responses to the reviewers’ specific comments.***

***Best regards,***

***Joao Guilherme Alves***

***- Corresponding Author -***

Reviewer #2: 2. For sample size calculation: You have not answer my question yet. The primary outcome is the presence of leg cramps, while the outcome used for calculating sample size is the secondary outcome. This is a substantial issue for your protocol. That means your current sample size might be only sufficient for analysis of your secondary outcome. Also, please provide your function for sample size calculation. The reference Currently the 20% dropout rate is a little bit large. And I do not find a 50% reduction in your referent papers. Please have a check.

***Answer: Thank you for this observation. You are completely rigth. Our sample size was calculated based on the frequency of leg cramps which is the parameter most used in trials assessing leg cramps interventions. Systematic reviews have used frequency of leg cramps as a p*rimary outcomes. Based on this we changed our primary outcome from the presence of leg cramps to frequency of leg cramps.**

**Function for sample size was determined by the software “Clinical.com” →“Statistics” → “Sample Size Calculator” → “View Power Calculations”:**

**N1={z1−α/2∗p¯∗q¯∗(1+1k−−−−−−−−−−−√)+z1−β∗p1∗q1+(p2∗q2k−−−−−−−−−−−−−−√)}2/Δ2q1=1−p1q2=1−p2p¯=p1+kp21+Kq¯=1−p¯N1={1.96∗0.57∗0.43∗(1+11−−−−−−−−−−−−−−−√)+0.84∗0.7∗0.3+(0.45∗0.551−−−−−−−−−−−−−−−−−−√)}2/0.252N1=60N2=K∗N1=60N1={z1−α/2∗p¯∗q¯∗(1+1k)+z1−β∗p1∗q1+(p2∗q2k)}2/Δ2q1=1−p1q2=1−p2p¯=p1+kp21+Kq¯=1−p¯N1={1.96∗0.57∗0.43∗(1+11)+0.84∗0.7∗0.3+(0.45∗0.551)}2/0.252N1=60N2=K∗N1=60**

**p_1_, p_2_ = proportion (incidence) of groups #1 and #2
Δ = |p_2_-p_1_| = absolute difference between two proportions
n_1_ = sample size for group #1
n_2_ = sample size for group #2
α = probability of type I error (usually 0.05)
β = probability of type II error (usually 0.2)
z = critical Z value for a given α or β
K = ratio of sample size for group #2 to group #1**

**The 20% dropout rate is really a little big. However, we lost to follow-up only two participants and we studied 66 pregnant women in each arm, this allowed us to reduce de drop out to 10% in our sample size calculation (60 + 6 = 66).**

**A 50% reduction was cited by Supakatisant C, Phupong V, reference number 16 (Oral magnesium for relief in pregnancy-induced leg cramps: a randomised controlled trial. *Maternal & Child Nutrition* 2015; 11(2):139–145), methods section, “The sample size calculation was based upon the 50% reduction in frequency of leg cramps in both groups obtained from Dahle *et al*.'s study (Dahle *et al*.** [**1995**](https://onlinelibrary.wiley.com/doi/full/10.1111/j.1740-8709.2012.00440.x#mcn440-bib-0002)**)”.**

3.For missing value: In your flwo chart, all 66*2 were included into your analysis, however, in your table 2 only 64 in treatment and 64 in control group were included into analyses. While actually, the overall number of placebo is 66. Please re-check your numbers. Many other inconsistences in your manuscript. For example, in tbale 1, 36 in Mg group and 30 in placebo group were currently employed; while in total, 68 were currently employed. In table 2, 27,2 should be 27.2. Please check your manuscript thoroughly.

***Answer: Sorry for this mistake. This number in the table 2 was corrected (Placebo 64 to Placebo 66). Table 1 was also checked and corrected. All the manuscript was completely reviewed.***

Reviewer #3: (No Response)

Reviewer #5: The authors aimed to evaluate to evaluate the effect of magnesium supplementation for the prevention of leg cramps in pregnant women.

According to information provided by the authors, the study was part of the Brazil MAGnesium trial and registered at ClinicalTrials.gov (Identifier NCT02032186). The registration refers to the Brazil MAGnesium trial, but neither the current study is referred to nor are the aims of the study defined as secondary outcomes. Therefore, the presented manuscript describes an unplanned secondary analysis of the above mentioned randomized trial Brazil MAGnesium trial. A secondary analysis should be regarded as an observational trial and should be identified explicitly as such.

***Answer: The study was now identified as an observational trial.***

The CONSORT checklist does not longer correspond to the presented study. The STROBE checklist would be more appropriate and will guide the authors to important aspects, which should be included in the description of the study. E.g. the consideration of potential confounders in the statistical analysis of observational data is necessary and should be added.

***Answer: The CONSORT check list was changed to STROBE checklist. This limitation was added in the discussion: 4) Because this study was observational, it could be prone to biases.***

One further aspect is, that randomization corresponds to the estimated sample size (2000 assigned to magnesium, 1000 assigned to placebo (with 2:1 allocation ratio) according to protocol on ClinicalTrials.gov) and not to the subsample of 132 patients (with an apparent 1:1 allocation ratio). This means, that the quality characteristics of randomization no longer apply. The study is merely an observational trial.

***Answer: Following your orientation the study design was changed to an observational study.***

The sample size of the initial study is based on the primary outcome (perinatal composite outcome). Therefore, the sample size and corresponding power is not adequate for the current outcome (presence of leg cramps) and puts the validity of the analysis and subsequent conclusion into question.

***Answer: We agree with you but a sub-sample size was calculated based on previous trials with interventions to reduce the frequency of leg cramps. This is supposed to offer robustness to our results.***

Apart from that, the “new” calculation is based on “50% reduction of leg cramps” and not on the variable defined as the primary endpoint (presence of leg cramps) so that the methodological basis of the study in itself is also questionable.

***Answer: The primary endpoint was changed to the frequency of leg cramps. The sample size was calculated based on 50% reduction of leg cramps. Presence of leg cramps was now considered as a secondary outcome.***

I am sorry to say, but from my point of view there are unsurmountable methodological deficits.

***Answer: Thank you very much for your analysis. We hope that as the study design was now changed to an observational trial you can review your position.***
